# Music Engagement and Episodic Memory among Middle-Aged and Older Adults: A National Longitudinal Analysis

**DOI:** 10.1093/geroni/igab046.2630

**Published:** 2021-12-17

**Authors:** Hillary Rouse, Gizem Hueluer, Mia Torres, Yan Du, Kyaien Conner, Hongdao Meng

**Affiliations:** 1 SiteRx / University of South Florida, TAMPA, Florida, United States; 2 University of Bonn (Germany); 3 University of South Florida, University of South Florida, Florida, United States; 4 School of Nursing, University of Texas Health Science Center at San Antonio, San Antonio, Texas, United States; 5 University of South Florida, Tampa, Florida, United States

## Abstract

Recent research suggests that engagement with particular activities, such as music, can influence age-related changes in episodic memory. However, it is unclear whether, and to what, extent music engagement is associated with the trajectory of episodic memory. The objective of this study is to examine how passive (i.e., listening to music) and/or active (i.e., singing or playing an instrument) music engagement influences episodic memory over a period of 12 years. Secondary data analysis of a sample (N=5095) of cognitively healthy adults from the Health and Retirement Study were used for this study. Linear mixed effects models were used to examine the independent effect of different levels of music engagement (i.e., low, medium, and high) on changes in performance on episodic memory tasks, while controlling for confounding factors. Compared to those with low engagement (i.e., neither listening nor singing/ playing an instrument), respondents who reported being engaged at the medium (i.e., either listening or singing/ playing an instrument) or high (i.e., both listening and singing/ playing an instrument) level performed 0.24 (p=0.003) and 0.52 (p<0.001) points better, respectively. We found evidence that music engagement attenuated the decline in episodic memory. The findings suggest that music engagement may be a protective factor against aged-related decline in episodic memory. Therefore, music engagement may offer a promising non-pharmacological intervention for dementia risk mitigation among community-living middle-aged and older adults. Future research should examine whether interventions to increase music engagement can affect the trajectories of aged-related decline in cognition in this large and growing population.

